# Effects of Nitrogen-Application Rate on Forage Oat Yield, Photosynthetic Characteristics, and Nitrogen-Use Efficiency

**DOI:** 10.3390/biology15212016

**Published:** 2026-11-06

**Authors:** Jiabing Yu, Chao Li, Mei Xiong, Tiefeng Yu, Weiyan Cheng, Feng Hao, Kai Gao

**Affiliations:** College of Grassland, Inner Mongolia Minzu University, Tongliao 028000, China

**Keywords:** nitrogen-application rate, forage oat, yield, photosynthetic characteristics, nitrogen-use efficiency

## Abstract

The effect of nitrogen (N) (0, 75, 150, 225 and 300 kg N ha^−1^, N0-N4) on the yield, photosynthetic properties and nitrogen-use efficiency of forage oat (*Avena sativa* L.) was studied in the Horqin Sandy Land in northern China. Mantel tests and chord diagrams were used to investigate the correlation of traits. Plant height, stem–leaf ratio, hay production, chlorophyll content and heading stage photosynthesis indices showed a unimodal response with highest values at N3 (225 kg N ha^−1^; *p* < 0.05). The dry matter yield at filling stage, net photosynthetic rate (Pn) at heading stage and total chlorophyll content (Total Chl) of N3 were 74.52%, 101.65%, and 62.18% higher than that of N0. The nitrogen contribution ratio (NFCR) of N3 reached 42.70%, which was 60.59% greater than that of N1. Nitrogen uptake efficiency (NUpE) was achieved at N3 that was 21.50% relative to N1.The N3 treatment showed the best comprehensive performance across agronomic, photosynthetic and nitrogen-use efficiency traits. In contrast, N3 achieved a favorable balance between yield and nitrogen use efficiency and can be regarded as a suitable nitrogen application rate for high-yield forage oat production in this region.

## Simple Summary

Forage oats are a major forage crop in the Horqin Sandy Land, which supports local animal husbandry. Although nitrogen fertilizer responses in oats have been reported for this region, few systematic studies have explored the link between forage yield production and leaf photosynthetic performance. This growing season field experiment included five nitrogen-application gradients. Our findings revealed that the forage yield, photosynthetic capacity and nitrogen-use efficiency measures were unimodal to the increasing supply of nitrogen. The maximum overall performance was achieved with the 225 kg N ha^−1^ treatment and it declined with the over-supply of nitrogen. The obtained results provide a practical recommendation on the management of nitrogen fertilizer in the local production of forage-oats. These findings should be confirmed by additional multi-year and multi-location field experiments.

## 1. Introduction

The Horqin Sandy Land in Inner Mongolia is a key region in Northern China and serves as the primary zone for livestock (cattle and sheep) rearing and forage cultivation [1]. Oat (*Avena sativa* L.) is grown within this region as a vital cereal and fodder plant; nevertheless, insufficient earth fertility plus weak moisture and nutrient holding abilities jointly restrict pasture production and standards [2]. Nitrogen is a vital macronutrient that determines crop productivity, contributing 30–50% of the global cereal yield increment and exerting direct regulatory effects on crop growth [3], development, and physiological performance [4]. The nitrogen demand for oat growth is primarily met through exogenous fertilizer supplementation, and rational nitrogen-application can significantly promote oat yield formation [5,6]. The appropriate N application rate for oat production varies by region, largely driven by differences in local environmental and climatic conditions. For instance, the agricultural yield of oats within Turkey was detected at 100–200 kg·ha^−1^ [7], while the maximum hay yield in achieved in Turkey was 3.2 t·ha^−1^ [8]. This regional variation suggests that a site-specific N recommendation for the Horqin Sandy Land is required, which is essential to guarantee high and sustainable oat production in this region.

Nitrogen (N) represents a vital component which controls photosynthesis within plants. As a crucial element within chlorophyll frameworks, nitrogen supply promptly affects the concentration of foliage chlorophyll and the productivity of photosynthesis catalysts, which consequently regulate the net photosynthetic speed [9]. Earlier studies show that the amount of N applied significantly influences the photosynthetic characteristics of oat flag leaves, as photosynthetic efficiency has a tendency to be unimodal, increasing and decreasing with the level of N availability [10]. The oat net photosynthetic rate and nitrogen photosynthetic utilization efficiency have been observed to be higher with the application of nitrogen, and are increasing [9]. Nitrogen-utilization efficiency serves as a vital metric for evaluating plant nitrogen absorption and mirrors the capacity for nitrogen integration, movement, and redistribution. An appropriate nitrogen supply can boost the crop nitrogen-use efficiency, but excess nitrogen reduces this characteristic [11]. Moreover, the overuse of nitrogen causes pollution of the environment, nitrogen saturation, the acidification of soils and the inhibition of plant growth [5].

Recent research on oats have focused on the adaptability of varieties [12,13], planting patterns [14], planting densities [15,16], and type and application regimes of fertilizers [17]. Nevertheless, the integrated relationships among nitrogen-application levels, photosynthetic attributes, nitrogen-utilization efficiency and agricultural output, along with their underlying mechanisms, remain insufficiently explored. However, the mechanism by which the level of nitrogen application regulates agronomic characteristics, photosynthetic efficiency and nitrogen uptake; the role of photosynthesis in mediating the effects of nitrogen on yield development; and what physiological constraints are behind the productivity inhibition caused by excessive nitrogen supply is not clear.

To address these issues, the present study investigated the forage oat variety ‘Baylor’,grown in Horqin Sandy Land, Inner Mongolia. Their agronomic characteristics and production were evaluated during the jointing and heading stages, including stages of filling, and photosynthetic physiological indicators were measured at the heading stage to systematically describe the trait responses to different levels of nitrogen application. Mantel tests and chord-diagram analysis were used to assess the hierarchical relationships and key driving processes between yield, photosynthetic characteristics and nitrogen use. These results give a theoretical foundation on how the application of nitrogen fertilizer can be enhanced in the local cultivation of oats.

## 2. Materials and Methods

### 2.1. Study Site

The investigation was conducted within the Agricultural and Animal Husbandry Science and Technology Demonstration Park of Inner Mongolia University for Nationalities, situated in the Horqin sandy area, Tongliao City, Inner Mongolia Autonomous Region (122°03′ E, 43°38′ N). It is located at an elevation of 182 m and has a temperate continental monsoon climate, characterized by an average annual temperature of 6.3 °C and a lowest temperature of −31.1 °C. Frost-free days vary from 130 to 160 days. The annual rainfall is usually 360–480 mm. The climatic data during the oat-growing season (April to July) are shown in Figure 1. The soil type at the experimental site was Arenosol. The basic physicochemical properties were as follows: soil organic carbon (SOC), 6.86 g·kg^−1^; available potassium (AK), 58.7 mg·kg^−1^; available phosphorus (AP), 11.26 mg·kg^−1^; alkali-hydrolyzable nitrogen (AN), 18.2 mg·kg^−1^; and a pH of 8.5. The research site was supplied with water, primarily in the form of natural rainfall and additional watering had to be provided and was conducted throughout the development period. The overall quantity of additional irrigation in the growing season was 120 m^3^·ha^−1^ and the hydrological input to every plot was equal.

### 2.2. Test Materials and Fertilizers

The test oat variety was ‘Baylor’, a late-maturing forage oat native to Canada, with a high yield and lodging resistance. The plant is sensitive to water and nutrient availability, and the seeds were acquired from Beijing Zhengdao Technology Co., Ltd. (Beijing, China). The experimental fertilizers were urea (N 46%), calcium superphosphate (P_2_O_5_ 12%), and potassium sulfate (K_2_O 50%) Sinochem Fertilizer Co., Ltd., Beijing, China).

### 2.3. Experimental Design

A single-factor randomized block approach was utilized, involving five distinct nitrogen fertilizer levels: 0 kg N ha^−1^ (N0), 75 kg N ha^−1^ (N1), 150 kg N ha^−1^ (N2), 225 kg N ha^−1^ (N3), and 300 kg N ha^−1^ (N4). Nitrogen was applied in two stages: 60% as a foundation fertilizer and 40% as a side dressing during the jointing stage. Fertilization during the jointing phase (25 May 2023) represented 40%; P and K fertilizers were applied as basal dressings in a single dose, the application rate of P_2_O_5_ was 100 kg·ha^−1^ (equivalent to 43.6 kg pure P ha^−1^), and the dosage of K_2_O was 120 kg·ha^−1^ (equivalent to 99.6 pure K kg·ha^−1^). This experimental design comprised three blocks and five nitrogen levels, resulting in 15 experimental plots. The plots were 25 m^2^ (5 m × 5 m) in size. Seeding was performed manually on April 15, 2023, utilizing a row interval of 20 cm, seeding rate of 150 kg·ha^−1^, and seeding depth of 3 cm. The plots were separated by a 100 cm buffer strip. Fertilization was followed by the irrigation of each plot and emergence irrigation was carried out when necessary. The other administrative strategies were identical except the different nitrogen fertilizer applications.

#### 2.3.1. Sampling and Measurement

Plant height, yield, and stem–leaf ratio were measured at the jointing (25 May 2023), heading (15 June 2023), and filling stages (1 July 2023), respectively. Photosynthetic indices were measured at the heading stage (15 June 2023).

#### 2.3.2. Plant Height Measurement

Thirty consistent oat specimens were randomly selected from each plot. The vertical dimension from the earth’s surface to the uppermost growing tip was determined utilizing a steel tape, and the mean value was calculated.

#### 2.3.3. Stem-to-Leaf Ratio Measurement

The collected specimens were physically divided into stalk and foliage portions. These materials were subsequently heat-treated at 105 °C for 30 min and dried in an oven(Shanghai Yiheng Scientific Instruments Co., Ltd., Shanghai, China) at 65 °C for 48 h to attain constant weight to be analyzed. The dry mass of the leaves and stems wastaken separately to obtain the stem–leaf ratio which was stem dry mass/leaf dry mass.

#### 2.3.4. Yield Measurement

Three plots (1 m × 1 m) were established in each site and all the oat plants in these plots were cut at ground level to be analyzed later. The original mass was measured after the removal of the debris with an electronic balance (Model BSA124S, Sartorius, Göttingen, Germany) whose precision was 0.1 g. These new specimens were then taken to the lab and thermally treated at 105 °C for 30 min, and dried in an oven at 65 °C for 48 h until they reached a constant weight. The yield was calculated using the following equation: yield per unit area (kg·ha^−1^) = dry mass of quadrat (g·m^−2^) × 10 (1 ha = 10,000 m^2^).

#### 2.3.5. Photosynthetic Parameters Measurement

Ten uniform oat samples were randomly selected from each plot for assessment during the heading phase. Evaluations were conducted on bright mornings between 9:00 and 12:00. The third completely developed leaf starting from the summit was measured via a LI-6400 transportable photosynthesis apparatus (LI-COR, Inc., Lincoln, NE, USA). The operating conditions inside the leaf cuvette were set to: PPFD = 1500 μmol·m^−2^·s^−1^, reference CO_2_ = 400 μmol·mol^−1^, flow rate = 500 μmol·s^−1^, and cuvette temperature at 25 °C.

The concentrations of chlorophyll a and b were evaluated using a 95% (*v*/*v*) ethanol (Tianjin Damao Chemical Reagent, Tianjin, China)-based colorimetric technique. Fresh, fully developed leaf specimens (0.2 g) were chopped into small pieces and transferred to 10 mL centrifuge tubes filled with 8 mL 95% (*v*/*v*) ethanol. These vessels were covered with aluminum foil to prevent light exposure and maintained in darkness at room temperature until the testing. The samples were periodically shaken a few times until the tissues of the leaves became entirely pale white (approximately 24 h). The obtained solution was centrifuged at 4000 r·min^−1^ for 10 min. The spectrophotometer (Model T6, Puxi General Instrument Co., Ltd., Beijing, China) was used to measure the absorbance of the supernatant at 663 and 645 nm with a blank of 95% ethanol. The classical formula was used to calculate the concentration of chlorophyll a, b and total chlorophyll [18].

#### 2.3.6. Determination of Total Nitrogen Content [19]

Ten consistently growing oat specimens were selected from each plot for sampling at the filling stage and the aerial components were collected, delivered to the laboratory, and dried in an oven until a stable mass was reached. The overall nitrogen content within these upper parts was measured using the semi-micro Kjeldahl technique following concentrated sulfuric acid treatment. The nitrogen-contribution ratio (NFCR), agronomic nitrogen-use efficiency (ANUE), nitrogen partial factor productivity (PFPN), and nitrogen-uptake efficiency (NUpE) were calculated using Equations (1)–(4), respectively.

Nitrogen contribution rate (NFCR) was calculated as follows:

(1) $$NFCR(\%)=\frac{Y_{N}-Y_{CK}}{Y_{N}}$$

Agronomic Nitrogen Use Efficiency (ANUE) was calculated as follows:

(2) $$ANUE\;(kg\cdot {kg}^{-1})=\frac{Y_{N}-Y_{CK}}{F_{N}}$$

Nitrogen partial factor productivity (PFPN) was calculated as follows:

(3) $$PFPN\;(kg\cdot {kg}^{-1})=\frac{Y_{N}}{F_{N}}$$

Nitrogen uptake efficiency (NUpE) was calculated as follows:

(4) $$NUpE\left(\%\right)=\frac{U_{N}-U_{CK}}{F_{N}}\times 100\%$$

where U (nitrogen uptake) = total nitrogen content in aboveground plant parts × aboveground dry weight of the plant.

Notation: $Y_{N}$ = yield in N-fertilized plot, $Y_{CK}$ = yield in control plot (no N), $F_{N}$ = N application rate, $U_{N}$ = N uptake in N-fertilized plot, $U_{CK}$ = N uptake in aboveground plant parts in control plot.

### 2.4. Statistical Analysis

Microsoft Excel 2021 (Microsoft Inc., Redmond, WA, USA) was used to sort the experimental data. IBM SPSS Statistics 27.0 (IBM Corp., Armonk, NY, USA) was used to construct a single-factor random block model using the general linear model univariate module to perform variance analysis. The nitrogen-application rate was set as a fixed factor, and the block was set as a random factor. Before statistical analysis, multiple subsamples in each cell were averaged to obtain cell-level data. Tukey’s HSD test was used to compare the mean values after treatment, with a significance level of α = 0.05. Multivariate statistical analysis was performed using R 4.3.1 (R Core Team, Vienna, Austria) software: Pearson correlation analysis, Mantel test, and correlation chord drawing were performed using N1–N4 processing data (N0 was not included in this part of the analysis because some nitrogen efficiency indicators could not be calculated); before the Mantel test chord plot, all indicators were standardized by Z-score in IBM SPSS Statistics 27.0 to eliminate the interference by different indicator dimension differences on the analysis and visualization results. Origin 2023 software was used to draw the histogram, and the error bars in the figure represent the standard error (SE).

## 3. Results

### 3.1. Plant Height

Figure 2a shows that the oat plant height initially increased and subsequently decreased with the increasing rates of nitrogen application at the various growth stages. N3 treatment always yielded the highest plant height and N0 treatment had the lowest. There was no significant difference between N2 and N4 at the heading and grain-filling stages, but N2 was significantly higher than N4 at the jointing stage (*p* < 0.05). The N3 regimen resulted in a plant height of 80.53% at the jointing stage, 46.51% at heading stage and 46.00% at grain-filling stage relative to N0.

### 3.2. Stem–Leaf Ratio

Nitrogen supplementation had a significant effect on the stem–leaf proportion of oat in all the stages of development as illustrated in Figure 2b. The reaction to raising the amount of nitrogen had a bell-shaped curve at the jointing, heading and grain-filling stages with the maximum points being attained under N3. The stem-to-leaf ratio was in the order of N3 > N2 > N4 > N1 > N0. Meaningful distinctions were identified between N0 and various other treatments at all developmental stages (*p* < 0.05). Relative to N0, N3 boosted the stem-to-leaf ratio by 17.11%, 12.88%, and 34.29% at the jointing, heading and grain-filling stages, respectively.

### 3.3. Dry Matter Yield

As depicted in Figure 2c, dry matter yield production increased with plant growth throughout every nitrogen regimen. The dry matter yield had a unimodal response to the nitrogen application in the jointing, heading and grain-filling stages and was at maximum with the N3 protocol. The yield of N3 was significantly higher than the yield of any other group (*p* < 0.05) and the general order of yields between treatments was N3 > N4 > N2 > N1 > N0. The N3 regimen boosted the dry matter yield by 77. 96%, 95. 66% and 74. 52% at the jointing, heading and grain-filling stages respectively compared to N0.

### 3.4. Chlorophyll Content

Figure 3a demonstrates the effect of nitrogen on the leaf chlorophyll content of oat plants at the heading stage; the addition of nitrogen had a significant positive effect on the leaf chlorophyll content. The unimodal distribution of chlorophyll a (Chl a), chlorophyll b (Chl b) and total chlorophyll (total Chl) showed the highest rate of nitrogen application at N3 treatment and decreased at high nitrogen concentrations. These N3 peak values were found to be significantly high as compared to the other groups (*p* < 0.05). The order of Chl a was N3 > N4 > N2 > N1 > N0; Chl b and total Chl were in the order N3 > N2 > N4 > N1 > N0. Compared to N0, the N3 intervention boosted Chl a, Chl b, and total chlorophyll by 59.38%, 56.42%, and 62.18%, respectively.

### 3.5. Photosynthetic Characteristics

As illustrated in Figure 3b, four photosynthetic metrics of oats were evaluated during the heading phase: net photosynthetic rate (Pn), stomatal conductance (Gs), intercellular CO_2_ concentration (Ci), and transpiration rate (Tr). Pn, Ci, Tr and Gs exhibited unimodal patterns along the nitrogen gradient and all reached their peaks under the N3 treatment, in the order of N3 > N4 > N2 > N1 > N0 for Pn, Tr and Gs. The peak values of Pn, Ci, and Tr under N3 were notably higher than those of the other treatments (*p* < 0.05). Compared to N0, N3 treatment boosted Pn, Ci, and Tr by 101.65%, 22.42%, and 81.16%, respectively. The Gs was significantly greater under N3 than those under all other treatments, with an increase of 348.9% relative to N0. The transpiration rate and Ci for N0 were markedly lower than those for N2, N3, and N4 (*p* < 0.05).

### 3.6. Nitrogen-Use Efficiency

Nitrogen fertilization had a significant effect on the nitrogen-use efficiency measures of forage oats (Figure 4). The nitrogen fertilizer contribution ratio (NFCR) and nitrogen-uptake efficiency (NUpE) was unimodal with the nitrogen-application continuum which initially increased and then decreased with the highest values at N3 regimen. NFCR and NUpE in N3 were 60.59% and 21.50% greater than that of N1. The agronomic nitrogen-use efficiency (ANUE) and partial factor productivity of nitrogen (PFPN) decreased with the increase in the level of nitrogen application and the maximum values were recorded under N1. NFCR and NUpE in N3 were notably superior to those in N1, N2, and N4 (*p* < 0.05). ANUE and PFPN under N1 were markedly higher than those under N2, N3, and N4 (*p* < 0.05).

### 3.7. Mantel Test for Correlation Between Agronomic, Photosynthetic and Nitrogen-Utilization Indexes of Oats

As illustrated in Figure 5, the Pearson correlation matrix demonstrated robust positive associations between the agronomic and yield-associated traits of forage oats. Plant height (PH), dry matter yield (DMY), and stem–leaf ratio (SLR) evaluated during the jointing, heading, and grain-filling phases were largely positively linked to each other (*p* < 0.01). Photosynthetic physiological measures taken at the heading stage were also very strongly correlated with agronomic yield measures. Plant height, dry matter yield, and stem–-leaf ratio throughout various developmental stages showed substantial positive correlations with heading-stage Pn, Tr, Gs, Chl a, Chl b, and Total Chl (*p* < 0.01). Chlorophyll levels were notably positively linked to Ci (0.01 < *p* < 0.05). Substantial positive correlations were detected among diverse photosynthetic variables, indicating the synchronized reactions in chlorophyll constituents and gas exchange metrics to nitrogen fertilization.

Nitrogen-utilization efficiency metrics exhibited divergent correlation trends, and the contribution rate of NFCR and NUpE were significantly positively correlated with agronomic traits at each growth stage and photosynthetic indices at the heading stage (*p* < 0.01). ANUE and PFPN were significantly negatively correlated with the above agronomic and photosynthetic indices (*p*< 0.01). Mantel test findings suggested that the nitrogen-use efficiency trait matrix was highly correlated with both the agronomic yield and photosynthetic trait matrices (*p* < 0.001). The photosynthetic trait matrix was significantly correlated with the agronomic yield trait matrix (0.01 < *p* < 0.05). The Mantel’s r corresponding to the nitrogen-utilization trait matrix was higher overall, indicating that nitrogen-utilization characteristics were the key factors regulating oat agronomic growth and yield.

### 3.8. Chord-Diagram Analysis of Traits of Forage Oats Under Different Nitrogen-Application Treatments

The chord diagram evaluation of diverse nitrogen-application levels and several forage oat metrics is presented in Figure 6. Regarding agronomic yield parameters, plant height, dry matter yield, and stem–leaf ratios throughout various developmental phases demonstrated more intense correlations with medium-and high-N regimes, exhibiting more distinct yield-related correlation features. The association-strength metrics of agronomic factors during the heading and grain-filling periods peaked under N2 and N3 treatments. Agronomic variables during the jointing phase were tightly linked to the low-nitrogen N1 regime. The association-strength figures of heading-stage photosynthetic metrics and chlorophyll indices attained their maxima for N2 and N3, whereas the contribution scales of photosynthetic indicators dropped markedly under low-nitrogen N1 and high-nitrogen N4. Regarding nitrogen-use efficiency measures, NFCR, NUpE, PFPN, and ANUE exhibited more significant reactions to low-N environments. The standardized values of nitrogen-use efficiency markers increased under the low-nitrogen N1 treatment, and these metrics served as crucial correlates of oat growth under low-N conditions. The N3 regimen generated the most robust synergistic links among agronomic, photosynthetic, and nitrogen-use efficiency attributes. The N3 regimen had the highest synergistic relationships between agronomic, photosynthetic and nitrogen-use efficiency traits. Under the high-N (N4) regimen, the association-strength scales of yield, photosynthetic, and nitrogen-use efficiency metrics declined simultaneously, mirroring the suppressed growth and development of forage oats.

## 4. Discussion

### 4.1. Effects of Nitrogen-Application Rate on Agronomic Traits of Forage Oats

Nitrogen is an essential macronutrient for vegetation, and its availability greatly limits plant development and biomass accumulation [20]. Forage oats are extremely responsive to nitrogen supply and nitrogen management can significantly improve primary crop characteristics, such as plant height, number of tillers [21], and promote the growth and development of oats. Plant height reflects the growth and development status of crops, which affects crop yield [22]. The findings in this study indicated that the oat plant height during the jointing, heading, and grain filling stages initially increased and then decreased with increasing nitrogen rate and attained its highest value along the experimental gradient at 225 kg N ha^−1^. Overuse of nitrogen suppresses normal growth and development of crops as has been observed by Islam et al. [6].

Our research revealed that the photosynthetic performance and dry matter yield of forage oat could be greatly enhanced by increasing the nitrogen fertilizer application rate, and there was a definite range in nitrogen application. This finding is consistent with the findings of Gao et al. [23] and Lin et al. [15]. The highest rate of nitrogen application that was achieved in this study is however different from theirs. Gao et al. [23] reported that the optimal nitrogen-application rate reached 375 kg N ha^−1^ under high planting density, while Lin et al. [15] obtained a high-yield nitrogen-application rate of 173 kg N ha^−1^ through the synergistic optimization of nitrogen rate and planting density. In this study, the single-factor optimal nitrogen rate of 225 kg N ha^−1^ was obtained under conventional planting density, which is more in line with local production practices and exhibits wider regional applicability. In contrast, Zheng et al. [24] reported that the optimal nitrogen-application rate for forage oat under conventional planting density in Northern Shanxi was 180 kg N ha^−1^. This difference is most likely to be attributed to the increased soil water and fertilizer holding capacity of Northern Shanxi resulting in a decreased best nitrogen-application rate as compared to Horqin Sandy Land. Furthermore, this study further confirmed that the response trend of oat dry matter yield to nitrogen addition remains consistent across different developmental stages. By introducing the Mantel test and chord diagram, this work breaks through the limitations of traditional correlation analysis. Through the coupled analysis of photosynthetic parameters, yield formation and nitrogen-use efficiency, the nitrogen fertilizer management system for forage oat in sandy soil is transformed from the traditional strategy of maximizing leaf photosynthesis to a new paradigm that synchronizes nitrogen fertilizer supply with crop nitrogen uptake dynamics. Padheriya et al. [25] also confirmed that the forage and dry matter yields of oats could be significantly increased within an appropriate nitrogen-application range. In this experiment, the yield of forage oat ‘Baylor’ increased significantly as the nitrogen-application rate increased from 0 to 225 kg N ha^−1^, whereas the yield showed a downward trend when the rate further increased to 300 kg N ha^−1^. This indicates a clear threshold for the promoting effect of nitrogen application on oat growth and yield, which is consistent with the findings of Wilson et al. [26] and Maral et al. [27]. In conclusion, appropriate nitrogen application is key to promoting the growth and yield of oats. Such factors, like variety, geographical location, climate and soil conditions affect the nitrogen content. Therefore, it is necessary to select the appropriate nitrogen-application rate in combination with regional environment, variety, and other conditions in production, considering the trade-off between yield and nitrogen-use efficiency, to achieve a high and stable yield of oats and efficient utilization of resources.

### 4.2. Photosynthetic Traits and Their Association with Yield Formation in Forage Oat Under Different Nitrogen-Application Rates

Photosynthesis is a key physiological process that regulates the transformation, fixation, and partitioning of nutrients in plants, which is the main pathway for energy supply and biomass accumulation. It is regulated by physiological status, weather, and agronomic management practices [28]. Photosynthetic efficiency is measured by the net photosynthetic rate, which determines stable and high crop yields [9]; Chlorophyll is a catalyst for photosynthetic reactions, and its concentration directly determines leaf photosynthetic efficiency [29].

Nitrogen fertilizer is an important means of regulating the photosynthetic physiological characteristics of oats. An appropriate nitrogen supply can promote chlorophyll synthesis in oat leaves and improve photosynthetic efficiency. The excessive use of nitrogen led to the inhibition of photosynthetic activity in the reverse direction [30]. Nitrogen availability is directly related to the alterations of photosynthetic physiological parameters of plant leaves [31,32]. Photosynthesis and chlorophyll content was measured in this study at heading stage of crop growth. This stage involved the application of basal fertilizer and top-dressing nitrogen. All plots were set to the desired nitrogen rate at the jointing stage. Findings indicated that nitrogen addition had a significant effect on increasing the chlorophyll content and photosynthetic physiological parameters of oat crops. Every metric initially rose and subsequently fell as the nitrogen-application amount increased, peaking in the 225 kg N ha^−1^ experimental group. Relative to the no-nitrogen control, the photosynthetic and chlorophyll indicators were markedly boosted under 225 kg N ha^−1^ nitrogen treatment, suggesting that appropriate nitrogen dosing could facilitate the production of photosynthetic pigments and enhance the photosynthetic physiology of leaves. Low-N conditions limit the photosynthesis of oats, while the nitrogen-application level continues to increase, and the photosynthetic and chlorophyll indices fall back, indicating that there is an obvious threshold for the regulation of nitrogen on the photosynthetic characteristics of oats.

The formation of organic matter and the accumulation of dry mass in forage oats are primarily derived from leaf photosynthetic activity, whose assimilation capacity is closely associated with material-accumulation efficiency [33]. This study revealed that after nitrogen fertilizer application exceeded 225 kg N ha^−1^, both the photosynthetic parameters and oat yield were simultaneously suppressed.

Studies on other oat varieties in Horqin Sandy Land also found that the efficiency of light energy conversion decreased under high nitrogen-application conditions, and high nitrogen caused the canopy closure of leaf groups and decreased photosynthetic performance in leaves, which in turn restricted the increase in yield [23,34]. Combined with the results of this experiment, nitrogen fertilizer modulates the dry matter accumulation and final yield of oats in close association with alterations in leaf photosynthetic physiological status. Mantel correlation analysis showed that chlorophyll and photosynthetic parameters at the heading stage were significantly positively correlated with plant height and hay yield at subsequent growth stages, supporting a close physiological association between heading-stage photosynthetic performance and the vegetative growth and yield formation of oats in the middle and late growth stages. The results of chord analysis also showed that the correlation between agronomic, yield, and photosynthetic indices was stronger under medium and high-N treatments, and the synergistic response level of multiple indices was higher under N3. Under excessive nitrogen application, the correlation strength of each index decreased, which was not conducive to the growth of oats.

Under 225 kg N ha^−1^ nitrogen application, the agronomic traits and photosynthetic characteristics of oats responded well to nitrogen considering the performance of nitrogen utilization, which could be used as the recommended nitrogen-application level for the cultivation of forage oat ‘Baylor’ in Horqin Sandy Land. The results by Shah et al. [35] were consistent with those of this study, which also proved that reasonable nitrogen application could improve the photosynthetic performance of leaves, promote dry matter accumulation, and increase yield.

### 4.3. Effects of Nitrogen-Application Rate on Nitrogen-Use Efficiency of Forage Oat

The efficiency of nitrogen use is a direct indicator of the use of nitrogen resources and the economic advantages of agricultural production [36]. In this study, a significant correlation was observed between the nitrogen-utilization index of oats and the agronomic traits and photosynthetic indices during each growth period. The results of the Mantel analysis showed that the nitrogen-related index Mantel’s r was generally high, indicating that nitrogen was the main factor regulating the growth and yield formation of oats. The contribution rate of nitrogen fertilizer and nitrogen absorption efficiency first increased and then decreased with an increase in the nitrogen-application rate, and the 225 kg N ha^−1^ treatment was at a higher level in this study than in previous studies. The agronomic efficiency of nitrogen fertilizer and partial factor productivity of nitrogen fertilizer decreased continuously with the increase in the nitrogen-application rate, indicating that the nitrogen utilization of forage oats in Horqin Sandy Land had an obvious threshold response to nitrogen-application level, which was consistent with the conclusion of Gao et al. [23]. The fertility retention capacity of aeolian sandy soil in Horqin Sandy Land is low. Studies have shown a potential risk of nutrient leaching under high-N application conditions in this area [37,38].

The agronomic efficiency and partial factor productivity of nitrogen fertilizer in the experiment decreased gradually with the increase in the rate of nitrogen application which was consistent with Wang et al. [39] and Han et al. [40]. Nitrogen-fertilizer control does not only affect the growth of oat hay yield, but also changes the nutritional level and quality of forage plants. Designing a field fertilization scheme, should take into account and consider all the yield factors, nutrition needs and forage quality. It also implies that the excessive use of nitrogen application reduces nitrogen-use efficiency. In low-N conditions, it is possible to increase yield and efficiency by adding more nitrogen fertilizer but excessive nitrogen fertilizer will limit the growth of yield. Therefore, coordinating yield and nitrogen-use efficiency is the key to nitrogen management of forage oats in Horqin Sandy Land. Combined with the results of this experiment, the recommended nitrogen-application range for forage oat planting in this region is 150–225 kg N ha^−1^, which can meet the nitrogen demand of oat growth and maintain a relatively high nitrogen-use efficiency.

This experiment mainly evaluated the agronomic indicators of the aboveground parts of forage oats but did not determine other related indicators, such as soil physicochemical properties. Moreover, this study utilized a single oat variety from a single site within the Horqin Sandy Land. Agronomic characteristics were evaluated during the jointing, heading, and filling phases, whereas photosynthetic and physiological metrics were collected exclusively at heading, which did not reflect whole-season dynamic changes. Thus, it is difficult to represent the possible variations that might occur among years, varieties, and different environmental conditions. Future studies should be expanded to include multi-year and multi-site experiments, including the measurement of soil physicochemical characteristics, conducting more systematic studies on various varieties under various ecological environments, and enhancing continuous dynamic monitoring throughout the entire growth period.

## 5. Conclusions

Based on the multidimensional findings of agronomic traits, photosynthetic physiological characteristics and nitrogen-use efficiency in this experiment, the 225 kg N ha^−1^ nitrogen treatment performed better overall with regard to plant height, photosynthetic and physiological characteristics, nitrogen-use efficiency, and forage yield among the five tested nitrogen-application rates. Beyond this point, the development, the nitrogen utilization and photosynthesis of oats decreased at the same time. Among the five examined nitrogen rates, the 225 kg N ha^−1^ regimen displayed the most excellent integrated performance under our experimental settings for forage oat ‘Baylor’ in the Horqin Sandy Land. Consequently, 225 kg N ha^−1^ has been suggested as the nitrogen-application level for forage oat ‘Baylor’ cultivation in Horqin Sandy Land. In actual agricultural practices, a nitrogen-application interval of 150–225 kg N ha^−1^ should be utilized to reconcile forage production and nitrogen-utilization efficiency. The findings of this research can be applied to sustain forage production and minimize the risk of nitrogen leaching into the environment when used in agricultural production, prevent issues such as luxury nitrogen uptake and photosynthetic efficiency due to excessive nitrogen application, and finally realize the synergistic improvement of forage oat yield and nitrogen-use efficiency in the target region.

## Figures and Tables

**Figure 1:**
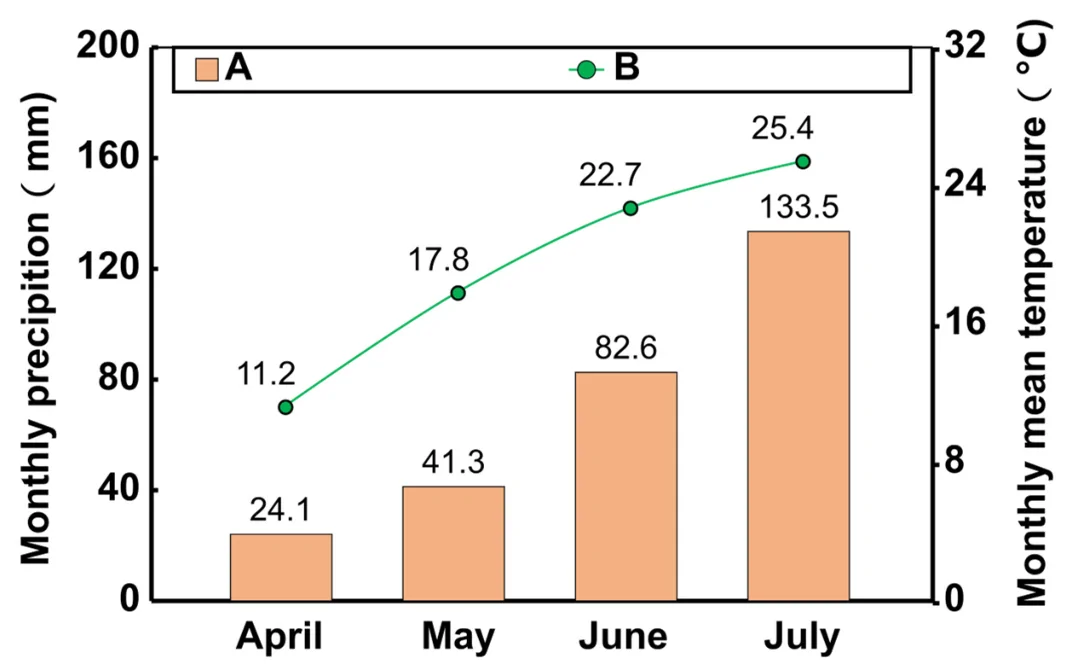
Mean monthly temperatures and precipitation levels during the cultivation phase of forage oats. Bars (A) represent monthly precipitation, and the line with solid circles (B) represents monthly mean temperature.

**Figure 2:**
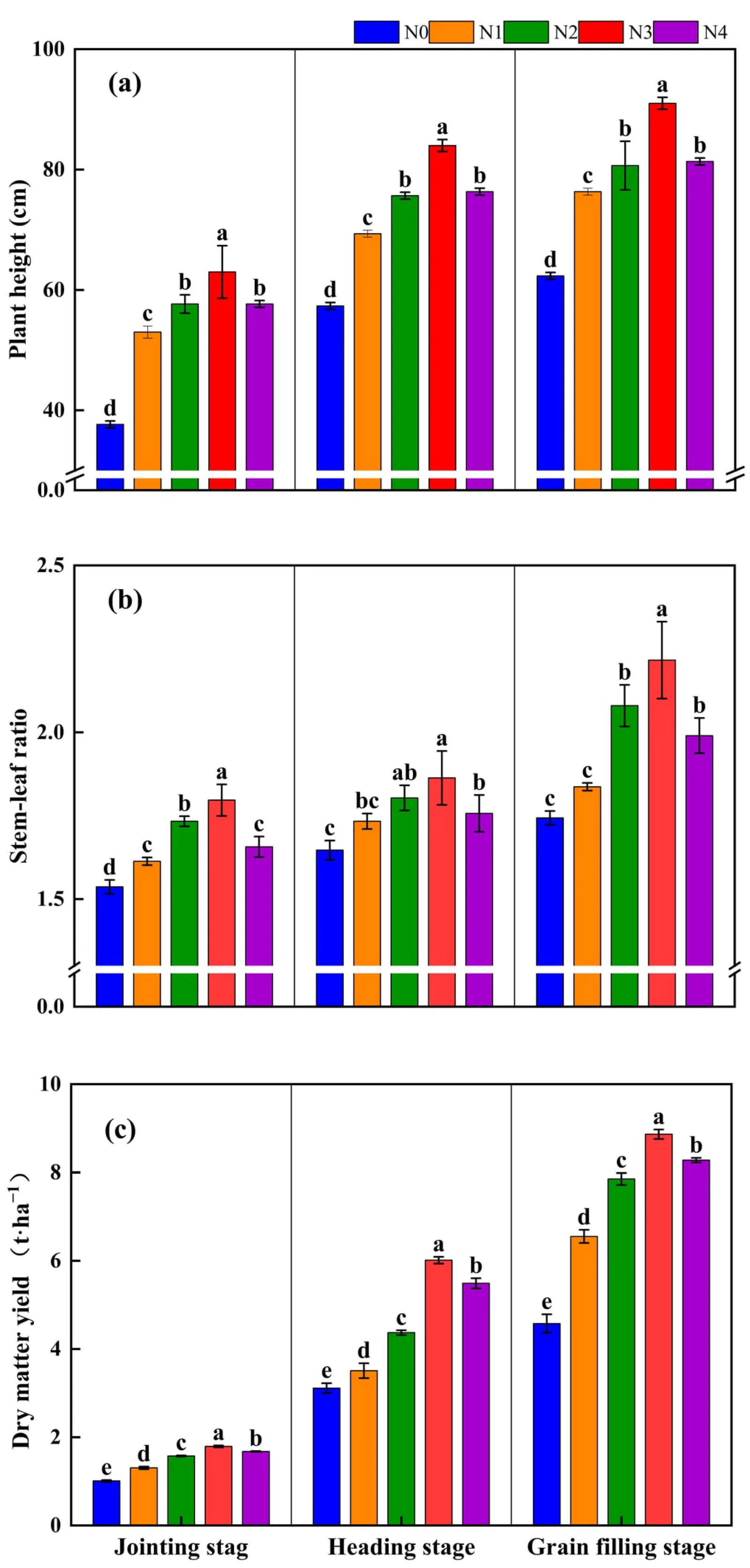
Effects of nitrogen-application rate on (**a**) plant height, (**b**) stem–leaf ratio, and (**c**) dry matter yield of forage oat at different growth stages. Note: Distinct lowercase characters denote statistically meaningful variations between various interventions (*p* < 0.05), identical to the following.

**Figure 3:**
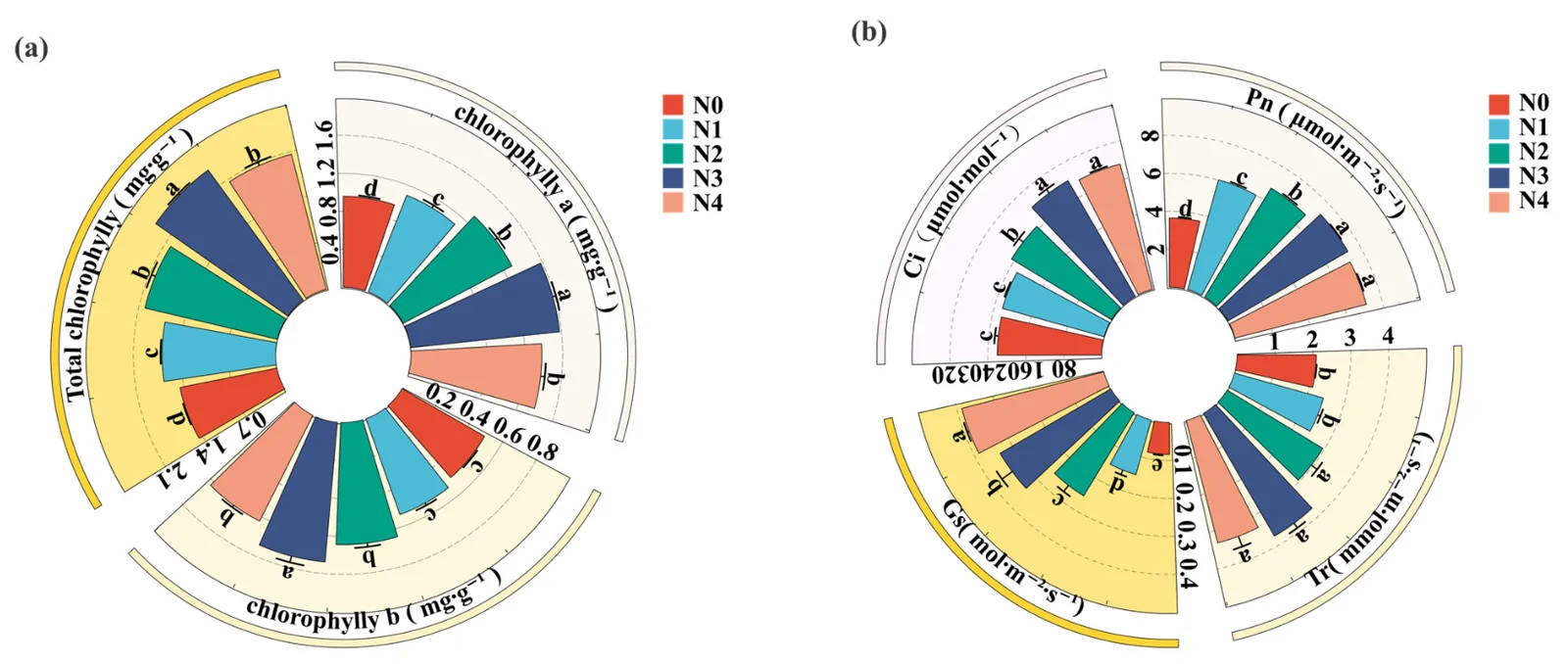
Effects of nitrogen-application rate on chlorophyll content and photosynthetic characteristics of forage oats. (**a**) Chlorophyll characteristics; (**b**) Photosynthetic gas exchange parameters. Note: Different lowercase letters indicate significant differences among treatments at *p* < 0.05. Different colors represent N0, N1, N2, N3 and N4 nitrogen treatments, respectively.

**Figure 4:**
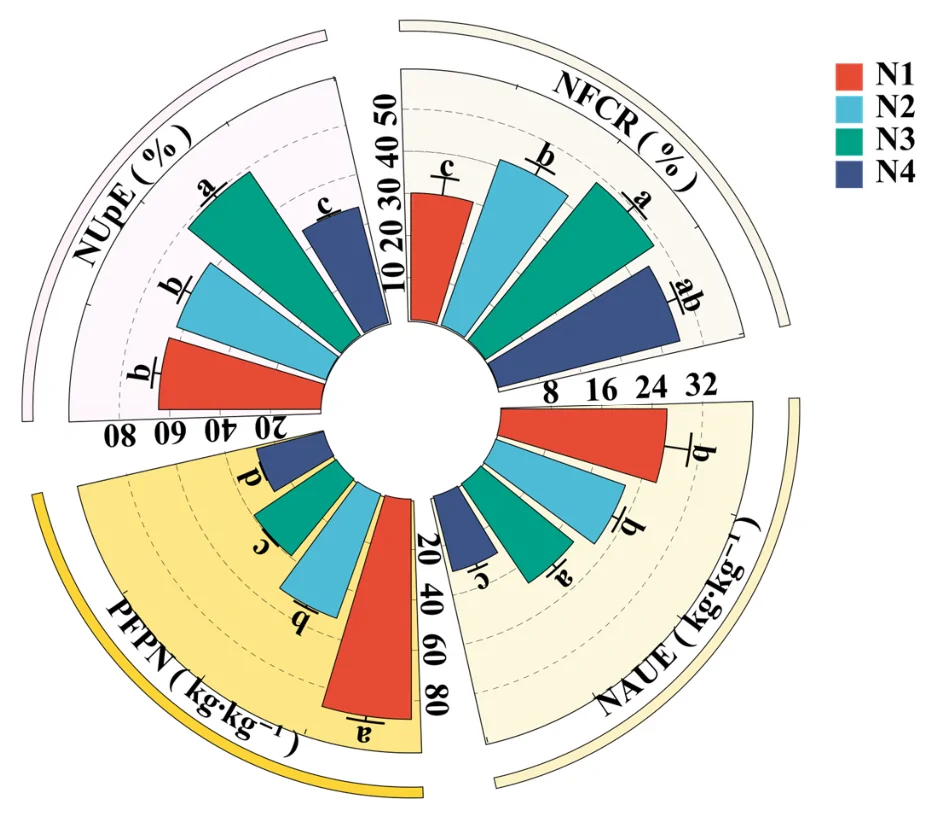
Effects of different nitrogen-application rates on the nitrogen-use efficiency of oats. Note: Different lowercase letters (a, b, c) indicate significant differences at *p* < 0.05. Different colors represent N1, N2, N3 and N4 nitrogen treatments, respectively.

**Figure 5:**
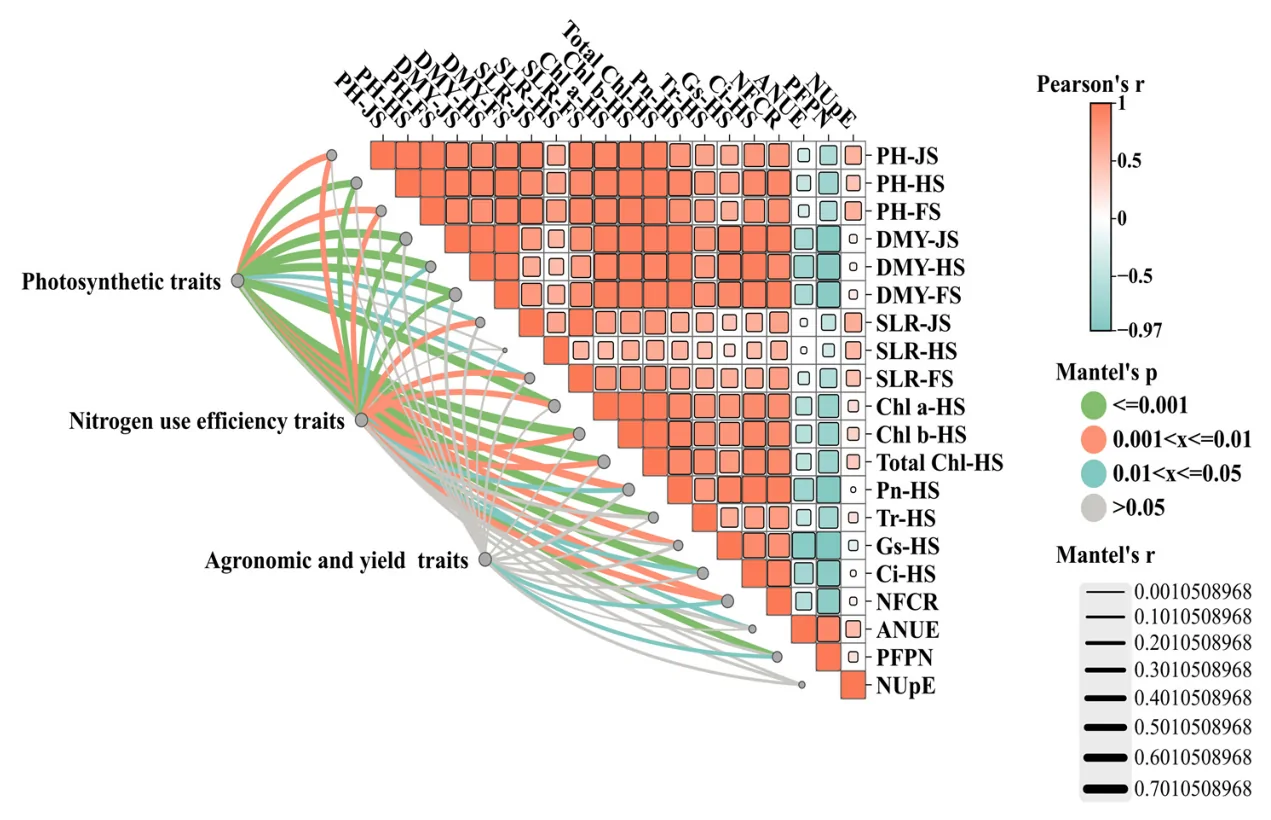
Mantel analysis of correlations among agronomic, photosynthetic, and nitrogen-use efficiency indicators of forage oat at different growth stages. Note: The right color block represents the Pearson correlation coefficient; the connection represents the Mantel test results between the different trait matrices. The color of the connection represents the significance level, and the thickness of the connection represents the size of Mantel’s r.

**Figure 6:**
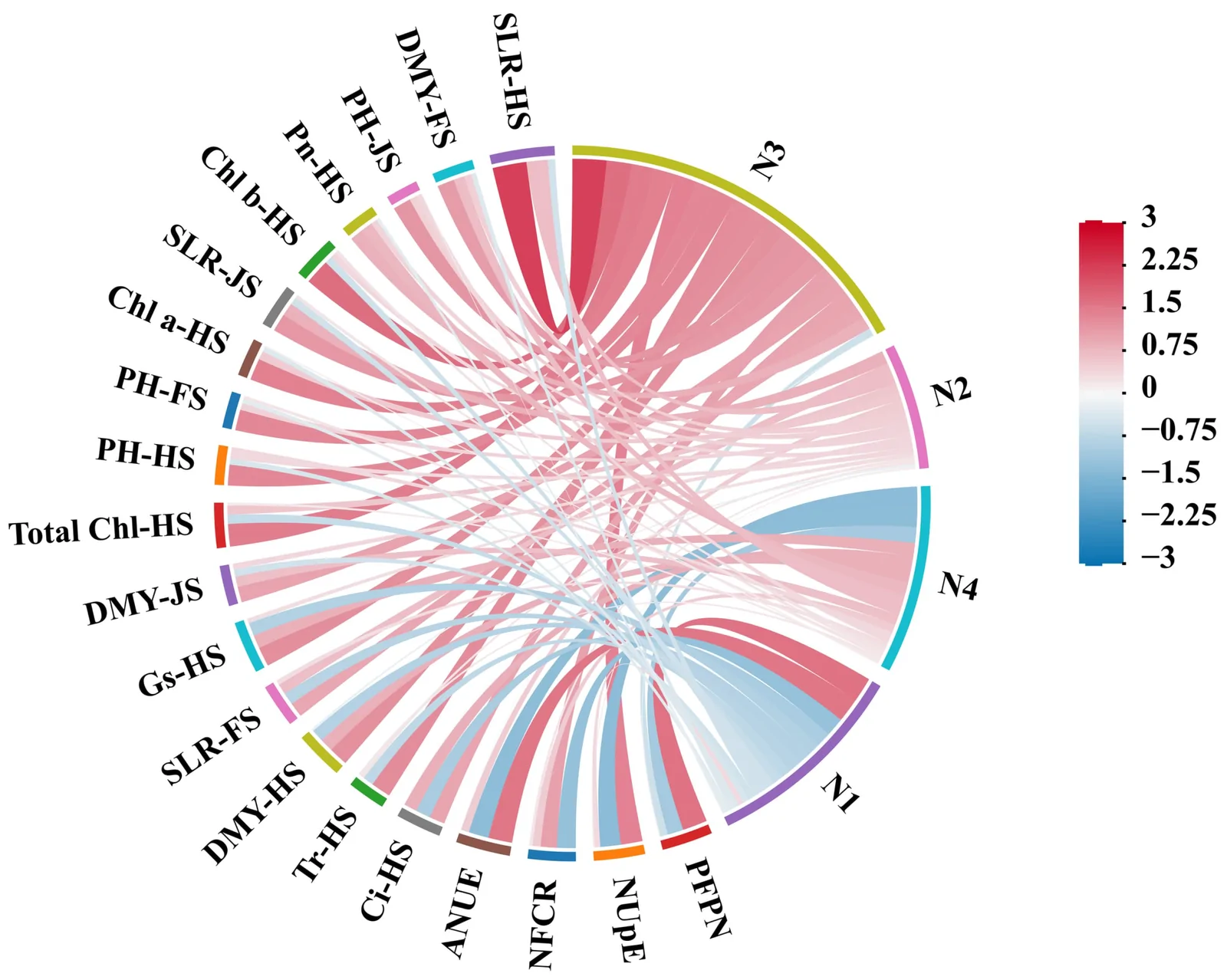
Chord diagram of each index of forage oat under different nitrogen-application treatments. Note: The outer ring node contains four nitrogen-application gradients (N1, N2, N3, N4) and various measurement indicators; the connection represents the correlation between the index and the nitrogen treatment, the color represents the standardized index value; the red represents a high value, and blue represents a low value.

## Data Availability

The original contributions presented in this study are included in the article. Further inquiries can be directed to the corresponding authors.

## Abbreviations

The following abbreviations are used in this manuscript:

N	Nitrogen
Pn	Net Photosynthetic Rate
Ci	Intercellular CO_2_ Concentration
Tr	Transpiration Rate
Gs	Stomatal Conductance
Chl a	Chlorophyll a
Chl b	Chlorophyll b
Total Chl	Total Chlorophyll
SOC	Soil organic carbon
AK	Available potassium
AP	Available phosphorus
AN	Alkali-hydrolyzable nitrogen
NFCR	Nitrogen Fertilizer Contribution Rate
NUpE	Nitrogen Uptake Efficiency
ANUE	Agronomic Nitrogen Use Efficiency
PFPN	Partial Factor Productivity of Nitrogen
PH-JS	Plant Height at Jointing Stage
PH-HS	Plant Height at Heading Stage
PH-FS	Plant Height at Grain filling stage
DMY-JS	Dry matter yield at Jointing Stage
DMY-HS	Dry matter yield at Heading Stage
DMY-FS	Dry matter yield at Grain filling stage
SLR-JS	Stem-Leaf Ratio at Jointing Stage
SLR-HS	Stem-Leaf Ratio at Heading Stage
SLR-FS	Stem-Leaf Ratio at Grain filling stage
Pn-HS	Net Photosynthetic Rate at Heading Stage
Tr-HS	Transpiration Rate at Heading Stage
Gs-HS	Stomatal Conductance at Heading Stage
Ci-HS	Intercellular CO_2_ Concentration at Heading Stage
Chl a-HS	Chlorophyll a content at Heading Stage
Chl b-HS	Chlorophyll b content at Heading Stage
Total Chl-HS	Total Chlorophyll content at Heading Stage
